# The Outcome of Decoronation in Severe Cases of External Cervical Root Resorption in Young Patients

**DOI:** 10.7759/cureus.62853

**Published:** 2024-06-21

**Authors:** Dina Moss, Eyal Nuni, Hagay Slutzky, Daniel Moreinos, Iris Slutzky-Goldberg

**Affiliations:** 1 Department of Endoodntics, Hebrew University Hadassah School of Dental Medicine, Jerusalem, ISR; 2 Department of Endodontics, Galilee College of Dental Sciences, Nahariya, ISR; 3 Department of Prosthodontics, Goldschleger School of Dental Medicine, Tel Aviv University, Tel Aviv, ISR; 4 Faculty of Medicine, Bar Ilan University, Sefad, ISR

**Keywords:** decoronation, external cervical resorption, dental trauma, orthodontic treatment, maxillary incisor

## Abstract

This study examines decoronation as a treatment option for teeth with progressive external cervical root resorption (ECR). Six young patients aged 9.5-13, with a total of nine incisor teeth affected by ECR due to previous dental trauma, were treated by decoronation. Six teeth were classified as class 4 and two as class 3, according to Heithersay's classification. Another tooth with class 2 resorption also had a perforation. After decoronation, all cases showed favorable outcomes during a follow-up period of 2.5-8 years. The procedure halted the progression of ECR and promoted vertical and horizontal ridge development above the submerged root. Decoronation can be considered for the successful treatment of advanced cases of ECR in young patients.

## Introduction

External cervical resorption (ECR) involves the periodontium, cementum, dentine, and pulp in later stages [1]. It usually starts at the cervical region of the affected tooth [2] and propagates in apical and coronal directions and around the canal without penetrating the pulp, which is surrounded by a peri-canalar resorption-resistant sheet [1,3]. The diagnosis of ECR may be challenging, especially in the buccal or palatal aspects of molar teeth. Only in the lesion's advanced stages might the root canal become perforated, leading to pulpitis and apical periodontitis [3]. The etiology of ECR is not fully understood. The protective cementum layer is assumed to be missing or damaged [3,4]. The resorption progresses in three stages: resorption initiation, resorption progression, and potential repair or remodeling. During the latter, bone-like tissue penetrates the resorption defect via the portals of entry and resorption tunnels, which might lead to tooth ankylosis [1,3,4]. Orthodontic treatment was suggested as a predisposing factor to the development of ECR, either as a sole factor, ranging from 24.1% to 45.7% [2-4], or as a combination of factors [2,4]. Dental trauma, especially avulsion and luxation injuries, result in localized damage to the attachment apparatus and are also associated with the development of ECR [4-6]. Treatment of ECR is based on the progression, extent, and location of the portals of entry. Small lesions can be treated surgically. However, in most cases, the resorption develops internally and circumferentially around the root canal and pulp. In such cases, endodontic therapy might be required [7]. Alternatively, orthodontic extrusion can enable an external approach to the resorptive lesion [2]. The root canal system might provide better access when surgical access is difficult, requiring significant bone removal [8]. Intentional replantation using a periotome or surgical blades and bioactive materials was also suggested [7,9,10]. In young patients, premolar auto-transplantation may be considered to replace a traumatized ankylotic maxillary incisor. For untreatable teeth, periodic follow-up is advised unless the tooth becomes symptomatic. Alternatively, atraumatic extraction of otherwise untreatable teeth can be considered [7]. Since the later phase of ECR is characterized by potential penetration of bone through the resorption channels [1], ECR, similar to replacement resorption, can cause infra-occlusion. The bone or bone-like tissue penetrating into the resorption defect may fuse with the dentinal structure of the teeth involved [1,10]. This may cause an esthetic and functional problem in young patients. Decoronation has been suggested for the treatment of infra-occluded incisors affected by replacement resorption caused by traumatic dental injuries [11,12]. This surgical procedure involves the removal of the crown and the coronal portion of the root to approximately 2 mm beyond the marginal bone, leaving the ankylotic root to be resorbed. Then, blood is allowed to fill the empty root canal. The blood clot that forms coronal to the remnant root will organize to form new bone. This technique enables the preservation of the alveolar ridge [13]. To the best of our knowledge, there have been no previous reports of successful decoronation in teeth with ECR. This study aimed to evaluate the treatment outcomes following the treatment of teeth with external cervical resorption and present decoronation as a possible procedure for teeth with progressive ECR.

## Materials and methods

A study was conducted on 50 teeth that were diagnosed and treated for ECR in 35 patients at the endodontic department. The cases were treated by postgraduate students between 2009 and 2018. Only cases with a minimum follow-up period of two years were included in the study. Nine young patients, aged 9.5-13, with severe ECR in nine incisor teeth underwent decoronation. Of the teeth with ECR, eight were maxillary central incisors, and one was a maxillary lateral incisor. Seven teeth lost vitality and were endodontically treated; six teeth were dressed with calcium hydroxide after trauma until planned decoronation; and one tooth underwent root canal treatment before ECR diagnosis. Three teeth that underwent severe intrusion had been orthodontically treated to extrude them immediately after the trauma. One tooth, replanted after avulsion, caused loss of anchorage during orthodontic treatment due to ankylosis.

The data collected for each patient included their demographics, general health, dental history, signs and symptoms, radiographic and clinical evaluations, stage of root development based on Nolla’s classification [14], stage of external root resorption according to Heithersay’s classification [5], follow-up period, and findings during follow-up. Additionally, each patient was asked about potential predisposing factors such as previous traumatic dental injury, orthodontic treatment, bleaching, and previous surgical procedures. The type and quality of the coronal restoration were also documented. Intraoral radiographs were taken using phosphor plates (MEDIADENT V6 Dental Imaging Software version© 6.12.6.20, The Dental Imaging Company, Ltd., Shoreham-by-Sea, UK) and Rinn XCP film-holding systems (Dentsply, Sirona, PA, USA). Teeth with ECR extending beyond the middle third of the root and showing signs of ankylosis were included in the treatment plan to receive decoronation.

Decoronation technique

The decoronation was carried out according to Malmgren's protocol [13]. Following administration of local anesthesia, 36 mg of 2% lidocaine, and 0.018 mg of epinephrine at 1:100,000, an intramuscular mucoperiosteal flap was raised. The crown of the tooth was separated from the root using a diamond bur, and the root was reduced to 2 mm below the bone level. A #35 endodontic stainless-steel Hedstrom file (Dentsply Sirona, Bern, Switzerland) was inserted through the root canal and beyond the apical foramen, and the canal was alternatively rinsed with saline to stimulate bleeding into the root canal. The flap was sutured with Vicryl 5/0 sutures (ETHICON TM, Bridgewater, New Jersey), which were removed five to seven days later. At the end of the surgical session, the patient received a prosthetic or orthodontic device in lieu of the removed crown, either as a trans-palatal arch orthodontic appliance (TPA), as a removable partial denture, or as an adhesive bridge restoration. The follow-up period ranged between 2.5 and eight years. Follow-up visits, including clinical and radiographic examinations, were scheduled after three and six months and then yearly.

Ethical and human considerations

This study was conducted in accordance with the Code of Ethics of the World Medical Association and was approved by the institutional ethics committee (HMO-19-0021). Written informed consent was obtained from the patient's parents before treatment.

## Results

All the treated teeth that were diagnosed with ECR and ankylosis had suffered previous traumatic injuries, including four avulsions, three severe intrusions, and two lateral luxations. The timing for each decoronation was determined based on the extent of infra-occlusion more than two years after the trauma. Two of the central incisors were classified as stage 9 according to Nolla 1960 [12,14] and the rest of the teeth as stage 10. The resorption was observed incidentally during routine follow-up examinations. The degree of resorption was classified according to Heithersay’s classification as follows: two class 3 cases, six class 4 cases, and one tooth classified as class

**Figure 1:**
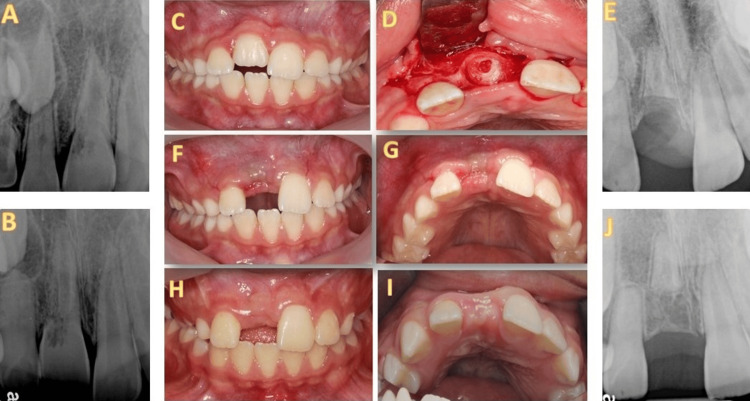
The successful decoronation of an intruded central incisor 30 months years after the treatment (A) Radiograph was taken a year after the intrusion of the upper right central incisor in a 9.5-year-old boy. ECR was observed on the distal aspect of the tooth, Heithersay's class 3. (B) ECR progression after six months. (C) Infraocclusion of the tooth is seen. (D) Photograph was taken during decoronation. The root was reduced to below the bone level, and the canal was allowed to be filled with blood. (E) Radiograph was taken immediately after decoronation. (F) and (G) One week after sutures removal. The flap had been tightly sutured. (H) and (I) Follow up 2.5 years after decoronation. Preservation of the width and height of the ridge can be demonstrated in horizontal (H) and vertical (I) aspects. The horizontal width is partially reduced compared to the adjacent tooth. (J) Radiograph demonstrating the horizontal ridge augmentation 2.5 years after treatment. PDL surrounds the submerged root. Pulp canal obliteration can be observed in the right lateral incisor, which can be related to a previous trauma (not reported).

In all cases, a favorable outcome was observed, including vertical and horizontal ridge augmentation coronal to the submerged root. The vertical aspect of ridge augmentation was based on a comparison of the postoperative and follow-up radiographs and measured in millimeters. The bucco-palatal assessment was based on a comparison to the contralateral tooth (Figure 1). A three-dimensional evaluation was postponed until implant placement was feasible.

**Figure 2:**
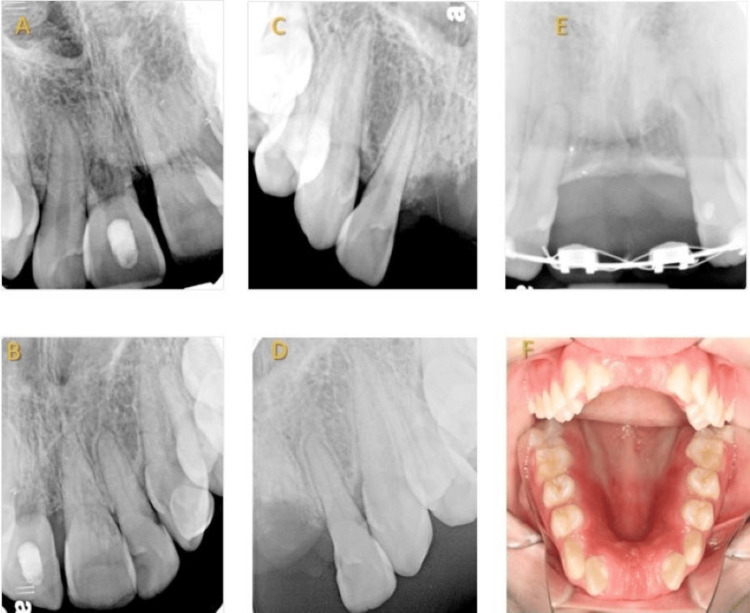
Decoronation of two adjacent maxillary central incisors A 10-year-old healthy girl presented two years after avulsion of the right maxillary central incisor and palatal luxation of the left central incisor. Ankylosis was demonstrated in both central incisors. (A) Preoperative radiograph demonstrating replacement resorption in the right central incisor. (B) External cervical resorption is observed on the distal aspect of the left central incisor. (C) Immediate post-operative radiograph after the decoronation of the right central incisor. (D) Immediate post-operative radiograph after the decoronation of the left central incisor. (E) A six-year follow-up demonstrating ridge augmentation in the horizontal aspect of the ridge. Similar results are observed in both teeth. ECR can be noticed in the distal aspect of the left maxillary lateral incisor. (F) A photograph of the anterior region demonstrating the full width of the ridge after the decoronation six years after the procedure.

Two patients had more than one incisor tooth decoronated. In one case, the two central maxillary incisors were decorated (Figure 2), and in another case, the treatment included the two central maxillary incisors as well as the left lateral maxillary incisors. After a follow-up period of four and six years, respectively, both treatments were considered successful.

## Discussion

The most commonly affected tooth by ECR is the maxillary central incisor, as found in previous reports [1,3,4,15]. The high incidence of ECR in maxillary incisor teeth is consistent with their frequent involvement in traumatic dental injuries [2]. Orthodontic treatment and dental trauma are the dominant predisposing factors for the occurrence of ECR. According to a study of 337 teeth, trauma and orthodontic treatment were the most common predisposing factors for ECR, accounting for 17% [4]. The resorption's multifactorial nature indicates the need for a more systematic approach to better understand the etiology [3-5]. All nine decoronated teeth treated by decoronation were maxillary incisors. In all the cases, dental histories revealed moderate to severe dental trauma, including luxation, intrusion, and avulsion. Four of the teeth had also been treated orthodontically. Replacement resorption and/or ECR often occur as serious complications following severe dental trauma [5,13].

Treatment options for more advanced stages of ECR lesions are limited to intentional replantation, atraumatic extraction, or periodic follow-up until the tooth becomes symptomatic, at which time extraction is required [7,10]. Intentional replantation of teeth not amenable to surgical repair is technique sensitive. Ankylosis, associated with the later fibro-osseous nature of the resorbing tissue, can cause infra-position of the teeth [11]. As a result of the ankylosis, the tooth might fracture during extraction. In some cases of intentional replantation, further resorption might occur at a later time [10]. In cases of replacement resorption caused by dental trauma, it has been suggested that ridge augmentation following decoronation would be advantageous if implant restoration is planned [11,15]. Six of the nine teeth treated by decoronation in this study had been diagnosed as class 4 and two as class 3, according to Heithersay's classification [5]. In one tooth classified as class 2, an additional root perforation was diagnosed during endodontic treatment. Accordingly, the prognosis was unfavorable; hence, decoronation was considered. One tooth was endodontically treated prior to the diagnosis of ECR. Calcium hydroxide dressing was placed in six teeth for its antibacterial properties until decoronation. It has been demonstrated that the sequelae of external cervical teeth in endodontically treated teeth are more severe than in vital teeth, probably because of the removal of the protective pulp and the peri-canalar resorption-resistant sheet during root canal treatment [1].

The patients scheduled for decoronation were between 9.5 and 13 years old. Malmgren has suggested that decoronation be performed before puberty to increase the likelihood of a favorable outcome [12]. Since the rate of infraposition of the ankylosed teeth is age-related [16], careful monitoring of the child's growth curve is required to achieve the best outcome [13]. Therefore, when considering decoronation as a treatment plan for patients with replacement resorption, timing has a significant effect on the outcome [13]. This applies whether the decoronation is performed as a sequel to a traumatic injury or in severe cases of ECR. Therefore, this treatment option is less suitable for adult patients because the outcome depends on the adjacent tooth's eruption [13,17]. In a study of 103 ankylotic permanent teeth, after decoronation, which was performed after the age of 16, the bone levels were unchanged or even decreased [18]. Therefore, other treatments, such as intentional replantation or extraction with or without implant placement, are necessary [8,10].

Upon clinical examination, all the teeth were found to be ankylotic. This could have been a result of severe dental trauma or the ingress of fibro-osseous tissue into the resorption defect [1,4]. Patel introduced a 3D classification in 2017 to describe the circumferential nature of resorption [9]. While a standard periapical radiograph may only show an ECR categorized as Heithersay’s class 3, a cone beam computed tomography (CBCT) scan has the potential to reveal a more widespread and severe Patel class 3Bp ECR. This type of ECR can be located subcrestally, extending into the middle third of the root and surrounding the root canal by 90º-180º. It may also affect the pulp, which could lead to an unfavorable prognosis [3] (Figures 1B, 2A). In 2018, the European Society of Endodontics recommended CBCT scans for teeth with ECR before deciding on a treatment plan [15]. If severe resorption is visible in the PA radiograph, i.e., Heithersay’s class 4, a CBCT scan is unnecessary as the tooth prognosis is unfavorable. Small-field CBCT is essential for diagnosing and following up on ECR cases, as it enables the clinician to visualize and quantify the stage of resorption [9]. Nevertheless, CBCT scans were not regularly conducted in the presented cases to avoid overexposure of children under 18 to radiation, in compliance with the AAE and AAOMR position statements regarding the use of CBCT in endodontics, which aim to maintain radiation levels as low as reasonably achievable [18]. Vertical ridge augmentation was observed in all the treated teeth. These results are comparable to the findings reported earlier on the decoronation of traumatized teeth after the occurrence of replacement resorption [11,12,19]. Although there was a minor decrease in the bucco-palatal dimensions of the ridge compared to the contralateral side, this is consistent with a previous study [20]. ECR is a progressive process. During the repair phase, a bone-like tissue resembling normal lamellar bone fills the resorbed defect, and the remodeling process begins [1]. This can explain the favorable outcome of decoronation in teeth with ECR.

In one case, decoronation of two adjacent maxillary central incisors was performed (Figure 2). The decoronation of the right central incisor was performed due to replacement resorption, following Malmgren’s classic indications [12], whereas the same procedure was carried out in the left maxillary incisor after a diagnosis of external cervical resorption. Nevertheless, there was a significant increase in the level of alveolar bone observed for both teeth during the six-year follow-up. These findings differ from a previous study, which discovered that when only the central incisors are decoronated, there is only resorption of the sharp edges of the bone between the teeth without an increase in bone height. Whereas better results are seen when both the lateral and central incisors are decoronated [21]. The positive outcome presented above was similar for both teeth, despite the different causes that led to the decoronation. This may imply that this procedure is suitable for teeth with replacement root resorption as well as external cervical root resorption.

Three options for restoring the missing crown after decoronation were suggested: a removable partial denture, a lingual orthodontic bar, or an adhesive bridge [16]. A future implant can be considered at the end of the skeletal growth [20]. However, even if the vertical dimension is preserved after decoronation, horizontal bone augmentation may still be necessary before implant placement [17]. At this stage, a follow-up CBCT scan before implantation would be mandatory. The remaining root may not be fully resorbed and is eventually translocated to a more apical position (Figure 1). Nevertheless, it was reported that the presence of root remnants after decoronation did not affect the outcome of implant placement and their integration [13], even when the implant was in close contact with the root dentin [22,23].

The appropriate treatment plan should be adjusted according to the severity of the ECR. Whereas intra- or extra-coronal approaches are suggested for the treatment of less extensive stages of ECR, decoronation can be considered in young patients as an alternative to tooth extraction in severe cases of ECR. The endodontic literature has shown the advantage of decoronation for future restoration [11].

Decoronation in young patients may pose some ethical concerns that should be considered: Information about the decoration procedure, its risks, benefits, and alternative treatment options must be delivered to the patient and guardian. This ensures fully informed decision-making. A special effort must be made to explain the removal of the crown and the necessity of providing an esthetic and functional interim prosthetic replacement. The anticipated timeframe for receiving a permanent restoration or until implant placement must be clearly explained [24,25].

Furthermore, decoronation can be challenging, especially for very young children. Behavior-management techniques are necessary to minimize anxiety and ensure cooperation [25]. Uncooperative patients or patients with special needs may require deep sedation or general anesthesia to complete the decoronation safely and efficiently [26].

Despite the success of the cases presented above, this procedure is limited to young patients under the age of 16 and may not be suitable for adult patients. The favorable outcome was demonstrated only in maxillary incisor teeth and was not tested in the mandible or in posterior teeth. A large-scale study is required to evaluate the potential use of decoronation as a treatment approach for teeth severely affected by external cervical resorption, as well as its applicability to other types of teeth.

## Conclusions

Considering the extent of root resorption, the prognosis in all the reported cases was unfavorable. Decoronation of teeth with external cervical resorption enabled the preservation of the alveolar ridge for future prosthetic rehabilitation by either an implant or a fixed appliance and prevented the formation of a rigid tissue effect. A comparison of the decoronation of two adjacent teeth with different diagnoses, either replacement root resorption or ECR, demonstrated a similar increase in the level of the alveolar bone. Due to the limited number of cases, a large-scale study must validate the current findings. In conclusion, decoronation can be considered for the treatment of teeth affected by ECR in young patients in their advanced stages.
